# Adaptation to unstable coordination patterns in individual and joint actions

**DOI:** 10.1371/journal.pone.0232667

**Published:** 2020-05-11

**Authors:** Thomas Wolf, Natalie Sebanz, Günther Knoblich

**Affiliations:** Department of Cognitive Science, Central European University, Budapest, Hungary; University of California Los Angeles, UNITED STATES

## Abstract

Previous research on interlimb coordination has shown that some coordination patterns are more stable than others, and function as attractors in the space of possible phase relations between different rhythmic movements. The canonical coordination patterns, i.e. the two most stable phase relations, are in-phase (0 degree) and anti-phase (180 degrees). Yet, musicians are able to perform other coordination patterns in intrapersonal as well as in interpersonal coordination with remarkable precision. This raises the question of how music experts manage to produce these unstable patterns of movement coordination. In the current study, we invited participants with at least five years of training on a musical instrument. We used an adaptation paradigm to address two factors that may facilitate producing unstable coordination patterns. First, we investigated adaptation in different coordination settings, to test the hypothesis that the lower coupling strength between individuals during joint performance makes it easier to achieve stability outside of the canonical patterns than the stronger coupling during individual bimanual performance. Second, we investigated whether adding to the structure of action effects may support achieving unstable coordination patterns, both intra- and inter-individually. The structure of action effects was strengthened by adding a melodic contour to the action effects, a measure that has been shown to improve the acquisition of bimanual coordination skills. Adaptation performance was measured both in terms of asynchrony and variability thereof. As predicted, we found that producing unstable patterns benefitted from the weaker coupling during joint performance. Surprisingly, the structure of action effects did not help with achieving unstable coordination patterns.

## Introduction

When humans engage in rhythmic joint actions, the underlying rhythm can act as a coordination smoother [1] and allows for especially tight temporal coordination. In rhythmic interactions, such as joint music-making, temporal coordination can reach a precision of a 100^th^ of a second [2]. Temporal coordination, however, is easier for certain coordination patterns than for others. In-phase coordination has been shown to be the most stable coordination pattern for intra- as well as for interpersonal coordination, followed by anti-phase coordination which is less stable [3–9]. These two coordination patterns have been called ‘canonical steady states’ [10]. This means that *unstable coordination patterns*, i.e., patterns other than in-phase or anti-phase, are especially challenging for temporal coordination, both during joint actions as well as during intrapersonal coordination of different limbs. Here, we will address two factors that could facilitate temporal coordination of unstable patterns. First, we will argue that despite extra effort being required during interpersonal coordination, adaptation to unstable patterns is actually more efficient during joint actions than during individual performance. Second, we will investigate whether the structure of action outcomes can provide a scaffold for achieving unstable patterns.

The requirement to produce an unstable coordination pattern, emerges for example, when three people stand around a large tent pole and use three hammers to drive the pole into the ground. The pole is too narrow to allow for in-phase coordination. To avoid collisions their timing has to be coordinated in a way that maximizes the time between each person’s stroke and the preceding and succeeding strokes. Since there are three people involved, the coordination is organized around a phase shift of 360 degrees / 3, which corresponds to 120 degrees and falls in the instable area between in-phase (0 degree) and anti-phase (180 degree). Whereas this may seem like a rare example of an interaction that requires an unstable coordination pattern, in the domain of music-making patterns like this are common. Musicians regularly master temporal coordination despite difficult coordination patterns both in bimanual solo performance as well as, interpersonally, in joint music-making.

Polyrhythms, for example, combine rhythms at non-integer multiples of each other such as 3:2 and 4:3 [11–13], and are used in a wide variety of music genres [14–21]. Furthermore, some instrumentalists need to produce phase shifted movements with different limbs due to the physical setup of their instrument. This is for example the case on the violin where a string has to be pushed down with the left hand before the bow is moved or the string is plugged with the right hand [22]. Pipe organs, another example, can exhibit delays of up to 150 ms [23] that vary according to pitch and may vary for different manuals and pedals. Coordinating tone onsets during music-making therefore entails compensating for these different delays by introducing offsets between instrumental actions. In mixed ensembles different instruments can exhibit distinct delays between movement initiation and sound onset [24,25]. Nevertheless, musicians have to coordinate their tone onsets by compensating for various delays.

### Previous literature

Early experiments on interlimb coordination found specific patterns of break-down suggesting that coordination of rhythmic limb movements is governed by the same laws as coupled oscillators [3,4]. When Haken, Kelso and Bunz [26] modelled interlimb coordination in terms of two coupled oscillators, coupling strength was taken to be an important parameter that governs how the coordination unfolds. The stronger the coupling the easier it is to maintain simple relations like in-phase coordination and the harder it is to maintain unstable coordination patterns.

Coupling strength influences both intrapersonal as well as interpersonal coordination. This has been shown for example in an interpersonal leg oscillating task by Schmidt and colleagues [6]. In a subsequent paper, Schmidt, Bienvenu, Fitzpatrick & Amazeen [27] found coupling to be significantly lower during interpersonal limb coordination than during intrapersonal limb coordination. In a more fine-grained visual coupling manipulation, Richardson et al. [5] compared peripheral visual coupling with direct visual coupling and found significantly more un-instructed in-phase coordination in the direct vision condition than in the peripheral vision condition, presumably due to the stronger coupling in the former, as weaker coupling reduces the tendency to fall into in-phase coordination.

As the coupling between two limbs of two individuals has been shown to be weaker than the coupling between two limbs of the same person [27], we argue that it is easier to distribute unstable coordination patterns among the limbs of multiple musicians. Ugandan xylophone music is a perfect example of how this can be exploited by composers and musicians. *Amadinda* music [28,29] is traditionally performed by three musicians on one instrument. Kubik describes the emergence of intricate *inherent rhythms*, which are “played at an incredible speed” [29]. These inherent rhythms however are not played by any individual musician, but instead shared across the first and second player in such a way that parts of the pattern that are in-phase are produced intrapersonally, whereas more complex phase relations are distributed interpersonally.

Whereas it might be easier to adapt to unstable coordination patterns in joint performances, musicians are able to perform unstable patterns both in joint and individual coordination settings. We therefore investigated a second factor that could facilitate adaptation across joint and individual performances. On a physiological level, the co-activation of homologous muscles has been proposed to account for the difficulties of producing certain bimanual patterns [4]. However, there is evidence for the claim that perceptual action effects, and especially their structure, plays an important role in enabling intra- and interpersonal rhythmic coordination [30].

Mechsner and colleagues [30] developed a task that isolated the effects of homologous muscles and perceptual symmetry of movements on rhythmic movement coordination. They found that perceptual symmetry was a better predictor for the stability of interlimb coordination. In another experiment Mechsner et al. [30] expanded this finding to a polyrhythm task, where musically untrained participants managed to produce circular motions in a 4:3 frequency ratio, when their goal was to perceptually align two rotating flags. Mechsner et al. took this as another piece of evidence that hand coordination is governed by perceptual features of action outcomes.

Researchers have studied similar facilitation effects in the auditory domain. Sonification, for example, can facilitate bimanual skill acquisition in the context of unstable coordination patterns [31]. Dyer and colleagues argued that these facilitating effects are caused by the perceptual unification of complex coordination patterns. The structure of auditory action effects seems to be an important factor for the unification. The same authors have shown that adding structure in the pitch dimension, i.e. adding a melodic contour, leads to faster skill acquisition, as it helps to better structure complex target patterns [32].

The role and strategies of the third player in Amadinda music is a good example of how musicians use auditory action effects to overcome motoric difficulties. The third Amadinda musician starts to play last and often has to execute especially complicated patterns. Kubik writes “it would be impossible to play this pattern […] by referring it metrically to one of the basic parts […]. The only chance to come in is by ‘thinking’ this pattern as a gestalt in its own right” [29]. Forming this Gestalt is facilitated by the fact that the pattern is already present in the joint outcome of the first two players [29].

Whereas all of the experimental studies reviewed so far used bimanual coordination in individuals, the Amadinda example and some studies of joint action suggest that there may be similar effects of action effect structure for joint performances. In particular, there is evidence that joint action is often based on representing joint action outcomes that combine individual action effects into a pattern that is more than the sum of its parts. Loehr, Kourtis, Vesper, Sebanz and Knoblich [33] invited pianists to duet with each other, while a computer introduced errors from time to time. An analysis of the performers’ EEG signals showed that it matters whether the introduced errors affected the joint outcome as much as the individual outcome. Aucouturier and Canonne [34] asked duos of improvising musicians to convey various social intentions and audio recorded their performances. Possible auditory markers of these social intentions were retrievable only from their combined audio signals, i.e. the joint outcome and not from any individual audio signal.

### Current study

Based on previous literature, we investigated two potential factors that may affect how able people are to produce unstable temporal patterns during rhythmic performances. First, we considered the inherent coupling strength characterizing different coordination settings. While the coupling between two limbs of the same person is strong, coupling between the two limbs of two people is weaker and the coupling between the limb of a person and a computer produced sounds is basically absent (unidirectional). Coupling strength is one of the factors that determines how difficult it is to maintain unstable phase relations [35]. To investigate effects of coupling strength as mediated by coordination contexts such as intrapersonal and interpersonal coordination, we devised an adaptation paradigm, where participants needed to align tone onsets triggered by finger tapping. In order to align tone onsets participants needed to adapt to an artificially introduced constant delay and thereby to an unstable coordination pattern. This coordination pattern required an interlimb phase relation of about 26.7 degrees. Based on previous literature, we expect participants to fail initially and therefore chose an adaptation paradigm to look at their improvement over time. If the coordination setting is indeed an important factor for the adaptation to unstable coordination patterns and this effect is mediated by coupling strength, we should see better adaptation in coordination settings with weaker inherent coupling than in coordination settings with stronger inherent coupling.

The second factor, structure of action effects, was tested in Experiment 2. Based on previous literature we examined the role of adding melodic structure to action effects. Experiments 1 provided participants with concurrent auditory feedback realized in percussive sounds to highlight the rhythmic pattern. In Experiment 2, we tested whether adding structure in the pitch dimension to the action effects helps to retain complex target patterns and thereby skill acquisition and performance [32]. To investigate whether similar effects occur independently of coupling strength, as the findings by Mechsner et al. [30] suggest, we tested this in the same coordination contexts as in Experiment 1.

## Experiment 1 –Methods

### Participants

Participants were recruited through flyers distributed in music schools in Budapest, on Facebook sites related to music-making in Budapest and on the CEU campus. Informed consent was obtained from all participants. Participants received gift vouchers as compensation. This study was approved by the United Ethical Review Committee for Research in Psychology (EPKEB) in Hungary. We invited 16 participants, of which 4 participants were not able to fulfill the minimal task requirements, i.e. they completed less than 80% of all trials successfully. The remaining 12 participants were included in the data analysis: 4 women, 8 men, mean age = 25 years, SD = 4 years. All participants in Experiment 1 had completed at least 5 years of private lessons on a musical instrument (M = 11 years, SD = 4 years). Sample size for both experiments was constrained by our ability to recruit musical experts with sufficient experience. Because this constraint implied a small sample size, we provide detailed information about statistical power to obtain large effects (Cohen’s f = 0.4), medium effects, (Cohen’s f = 0.25) and small effects (Cohen’s f = 0.1). We used G*Power (version 3.1) [36] to perform all power calculations. The resulting power for all within-subjects factors reported in Experiment 1 corresponds to 0.82, 0.41 and 0.10 for large, medium and small effects. These values are the same for the power in Experiment 2 because sample size and experimental design were the same.

### Apparatus and material

Participants were tapping on iPad Pros that were connected via the iOS app MIRA 1.2.2 to a custom patch created in Max MSP 7.3.1 running on a Mac computer. The Max patch created tap contingent sounds that were played back to the participants via headphones. An occluder prevented both participants from seeing their own iPad and their partner’s iPad in the Interpersonal condition.

### Procedure and design

**Fig 1:**
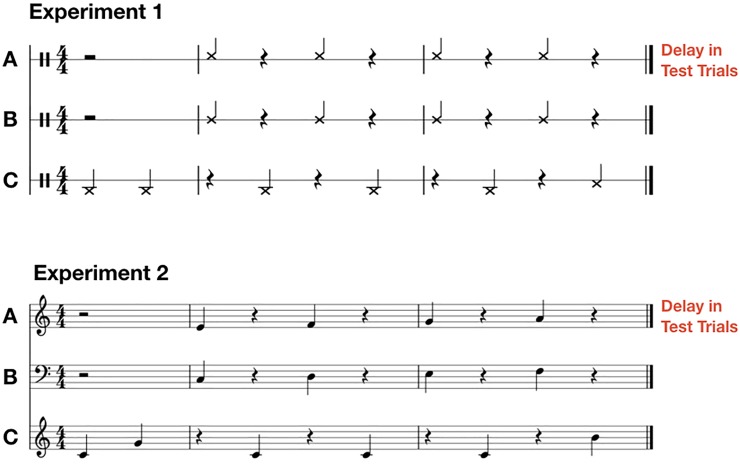
Sheet music representation of the target rhythm. In the Intrapersonal condition, staff A was always played by a participant’s right hand, whereas staff B was played by the same participant using her left hand, while the computer filled in staff C. In the Interpersonal condition, staff A was played by the participant on the right, while staff B was played by the participant on the left, both using their right hands. Staff C was again filled in by the computer. In the Computer condition, staff B and C were filled in by the computer, while the participant used her right hand to play staff A. This was the same for Experiment 1 and 2. During test trials the tone onsets of notes in staff A were artificially delayed by a Max patch.

Participants were instructed to tap out the rhythm depicted in Fig 1 at an inter-tap-interval (ITI) of 1200 ms. Two metronome clicks were followed by four finger taps of the participant. Four additional metronome clicks subdivided participants’ taps to assure a steady tempo. The last metronome click was realized as a bell-like sound to signal the end of the trial. In the Intrapersonal condition (high coupling), participants produced the rhythm alone using both hands, with one hand producing one percussion sound and the other hand producing a different percussion sound. Participants were asked to synchronize the two sounds produced by their two hands. In the Interpersonal condition, the two sounds needed to be synchronized across the two right hands of two participants, with the computer fulfilling the same metronomic function. For the Interpersonal condition the 12 participants were therefore grouped into 6 pairs. In the Computer condition, the computer provided the metronomic structure, but also produced sounds to synchronize with (like one’s other hand in the Intrapersonal condition and one’s partner in the Interpersonal condition). In this case the participant produced one percussion sound just as in the Interpersonal condition but with the computer as a perfectly timed partner.

In test trials, we introduced an artificial delay of 89 ms to the production of the sounds notated in staff A (see Fig 1). Hence, in order to align tone onsets, participants needed to compensate for this delay. The delay corresponded to 26.7 degrees of the 1200ms interval in phase space, a non-standard phase relation between two sound-producing movements.

**Fig 2:**
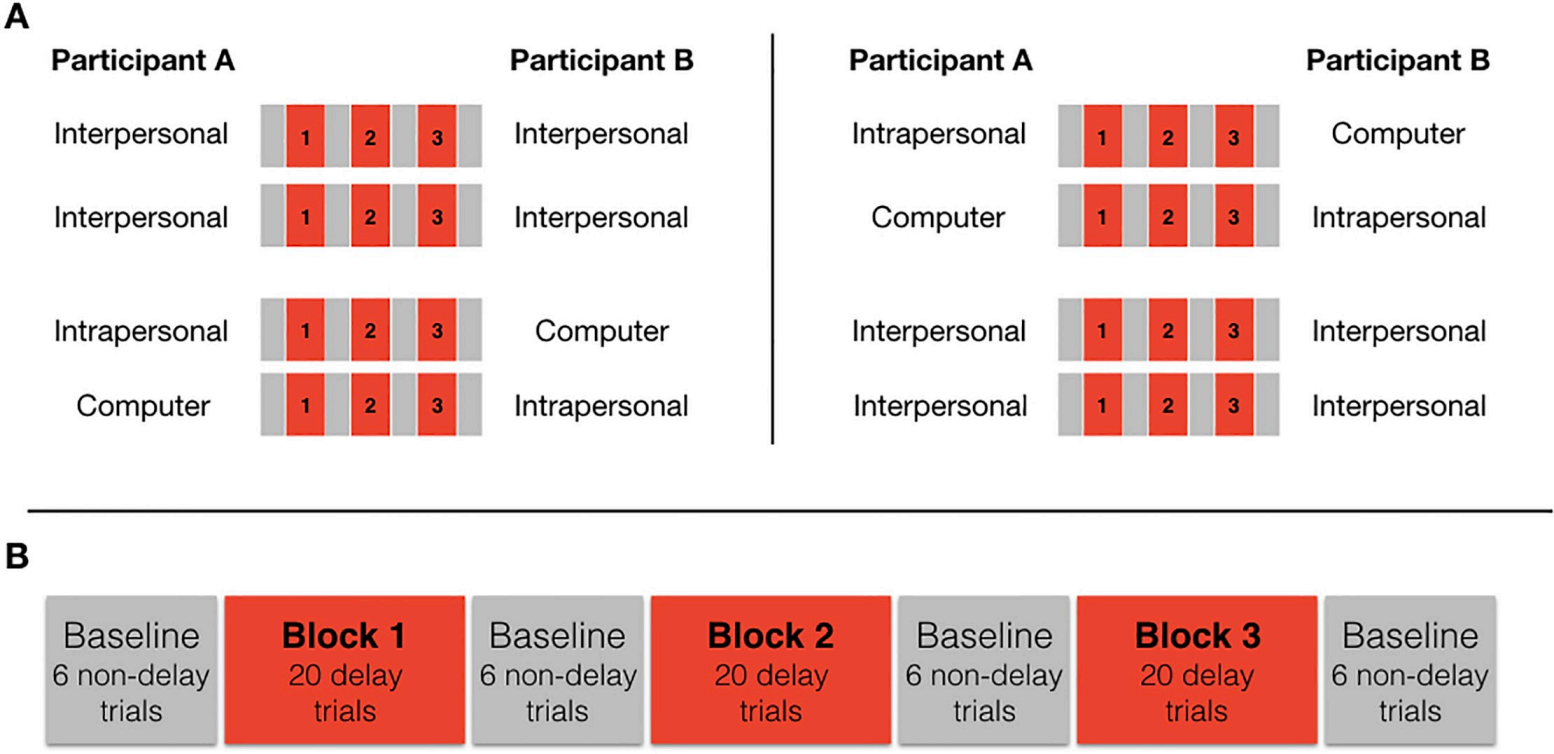
Procedure. Panel A) Each dyad completed four segments. Participants either started with two segments of the Interpersonal condition and then proceeded to individually complete one segment each of the Intrapersonal and the Computer condition (Panel A–left), or they started with the Intrapersonal and the Computer condition, before completing two segments of the Interpersonal condition (Panel A–right). In one of the Interpersonal segments, participant A experienced the delay and in the other segment participant B experienced the delay. Panel B shows the trial arrangement within one segment, which was the same in all conditions/throughout all segments.

Both experiments consisted of four segments (see Fig 2 –Panel A): Two segments involved joint performances (Interpersonal condition) and two segments were performed individually (Intrapersonal and Computer condition). Each pair of participants started either with the two segments in the Interpersonal condition or the two segments in the Intrapersonal and Computer condition. Who played which staff in the Interpersonal condition, and thereby also who experienced the delay, was switched after the first Interpersonal segment, so that each participant performed the Interpersonal condition playing staff A in one segment, which included the delay in some trials, and staff B in the other segment.

Each segment consisted of 24 baseline trials and 60 delay trials in the following arrangement (see Fig 2 –Panel B): 6 non-delay baseline trials; 20 delay trials (Block 1); 6 non-delay baseline trials; 20 delay trials (Block 2); 6 non-delay baseline trials; 20 delay trials (Block 3); and 6 non-delay baseline trials. This trial arrangement allowed us to collect baseline data in non-delay trials and to assess changes in performance over the time of 60 delay-trials. Participants were told in advance how many trials were coming up and whether or not they would include the delay.

### Analysis

Performance measures were computed from the timestamped midi data. First, we checked whether participants followed the task of producing the required rhythm. The interspersed metronome clicks allowed us to calculate target times for the tone onsets produced by the participants. We computed the temporal deviation from these target times. In the Intrapersonal condition this measure was computed for each hand, in the Interpersonal condition, for each participant and in the Computer condition only for the participant’s right hand. If a tap fell outside a target window of +- 300 ms around the target time, it was considered an error. A missing tap or too many taps between two metronome clicks were also considered to be errors.

The main dependent variables were absolute asynchrony and its variability. Absolute asynchrony between tone onsets in milliseconds is a direct measure of how well participants managed to align two sounds in time. Variability of absolute asynchrony, calculated as the standard deviation of absolute asynchrony also in milliseconds, describes how stable this temporal alignment is. We expected participants to show different baseline performances in different coordination settings. To account for this, we computed baseline corrected values for all asynchrony measures. For this calculation we took the asynchrony values and subtracted the condition-specific baseline performance for each participant. A baseline corrected value of around zero milliseconds therefor implied that the performance in the delay trials was equal to the performance in the non-delay trials.

To investigate whether participants’ group average improved over time, we computed 3 x 3 ANOVAs, with the two within-subjects factors Condition (Intrapersonal, Interpersonal and Computer) and Block (One, Two and Three). Significant results for Block imply a significant change of performance over the course of three blocks of delay trials. ANOVAs were computed for asynchronies and variability thereof in absolute terms and relative to baseline. Although the latter is more relevant for our study, we also included the former to establish comparability of our results with the results of previous studies addressing temporal coordination.

To assess whether individual participants managed to reach their baseline performance, we split each of the three blocks of delay trials in each condition into two halves which results in six bins spanning ten trials each. We then calculated 95% confidence intervals for mean absolute asynchrony and its variability for each of the 6 bins. Using both measures, asynchrony and variability, we determined in how many bins the non-delay baseline was encompassed by the confidence interval, i.e. in how many bins the performance on delay trials was not significantly different from baseline performance.

## Experiment 1 –Results

### Accuracy

Trials that included missing, additional or misplaced taps were marked as errors. These trials were excluded from further analysis. The mean accuracy for the participants in Experiment 1 was 93.49% (SD = 4.32%). A Greenhouse-Geiser corrected one-way ANOVA with the factor Condition (M_Intrapersonal =_ 93.35%, SD_Intrapersonal =_ 4.85%, M_Interpersonal =_ 90.58%, SD_Interpersonal =_ 8.24%, M_Computer_ = 96.53%, SD_Computer_ = 4.69%) revealed that the main effect of Condition fell just above a significance level of .05, *F*(1.531, 16.845) = 3.707, *p* = .056, *η*^*2*^ = .146. Hence, there was no significant difference among the conditions in terms of accuracy.

### Asynchrony in baseline trials

**Fig 3:**
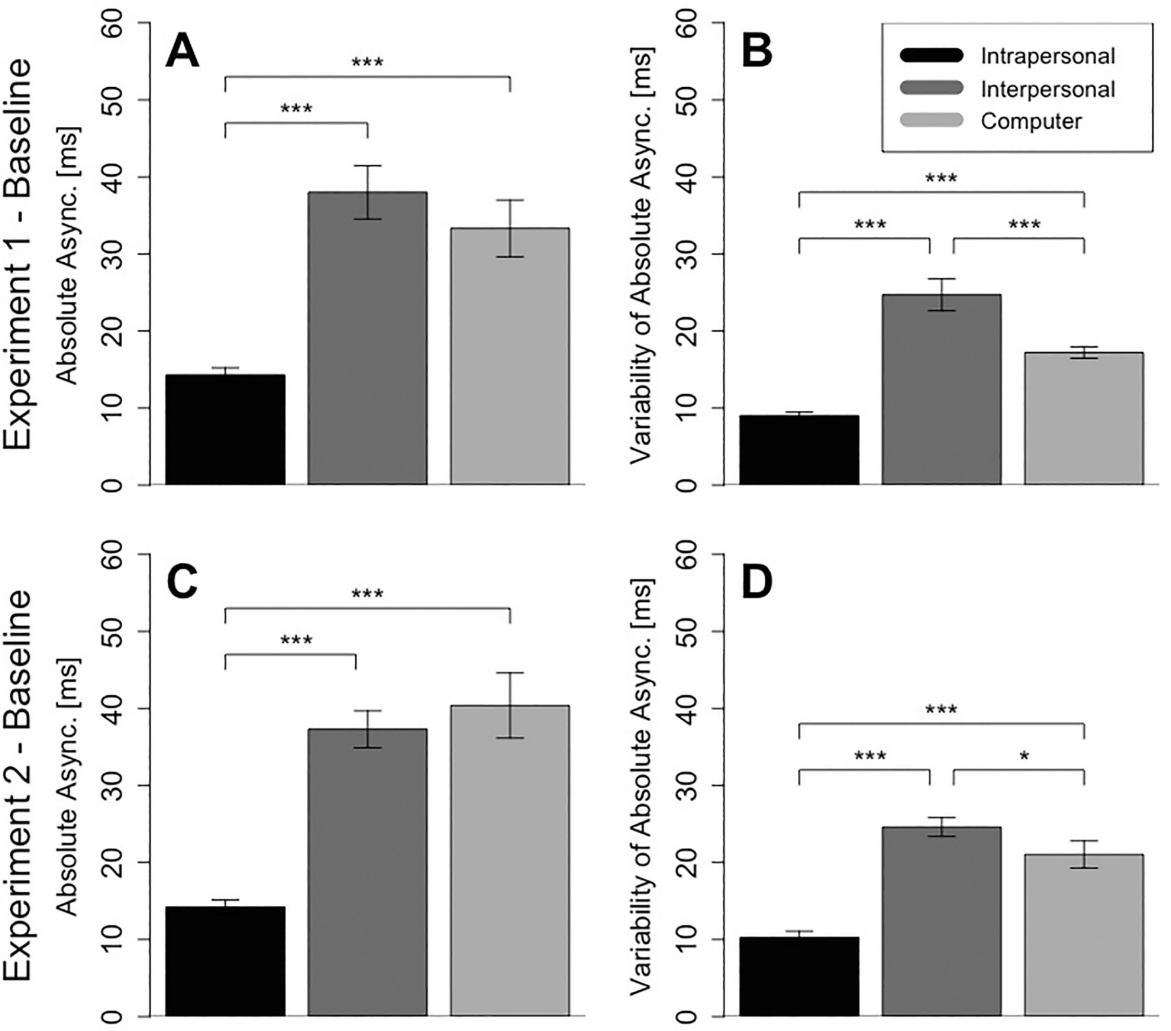
Baseline performance from non-delay trials for Experiments 1 and 2. The panels on the left (A and C) show absolute asynchronies and the panels on the right show standard deviation of absolute asynchronies (B and D). Error bars display standard errors in each condition.

For the performance in non-delay baseline trials we observed the lowest absolute mean asynchrony for the Intrapersonal condition (M = 14 ms, SD = 3 ms), with Joint (M = 38 ms, SD = 12 ms) and Computer (M = 33 ms, SD = 13 ms) showing comparable levels of asynchrony (see Fig 3 –Panel A). We computed a one-way ANOVA for the factor Condition (Intrapersonal, Interpersonal and Computer). The Greenhouse-Geiser corrected ANOVA revealed a significant effect for Condition, *F*(1.669, 18.356) = 18.963, *p* < .001, *η*^*2*^ = .520. Post-hoc paired-samples t-tests with Holm-Bonferroni corrected p-values [37] showed that this was mainly due to the lower absolute asynchrony in the Intrapersonal condition, as compared to the Interpersonal, *t*(11) = 6.613, *p* < .001, *d* = 1.909, and the Computer condition, *t*(11) = 5.277, *p* = .001, *d* = 1.523. There was no significant difference between the Interpersonal and the Computer condition *t*(11) = .955, *p* = .360, *d* = .276. To summarize, participants exhibited the best baseline performance in the Intrapersonal condition.

### Asynchrony in delay trials

**Fig 4:**
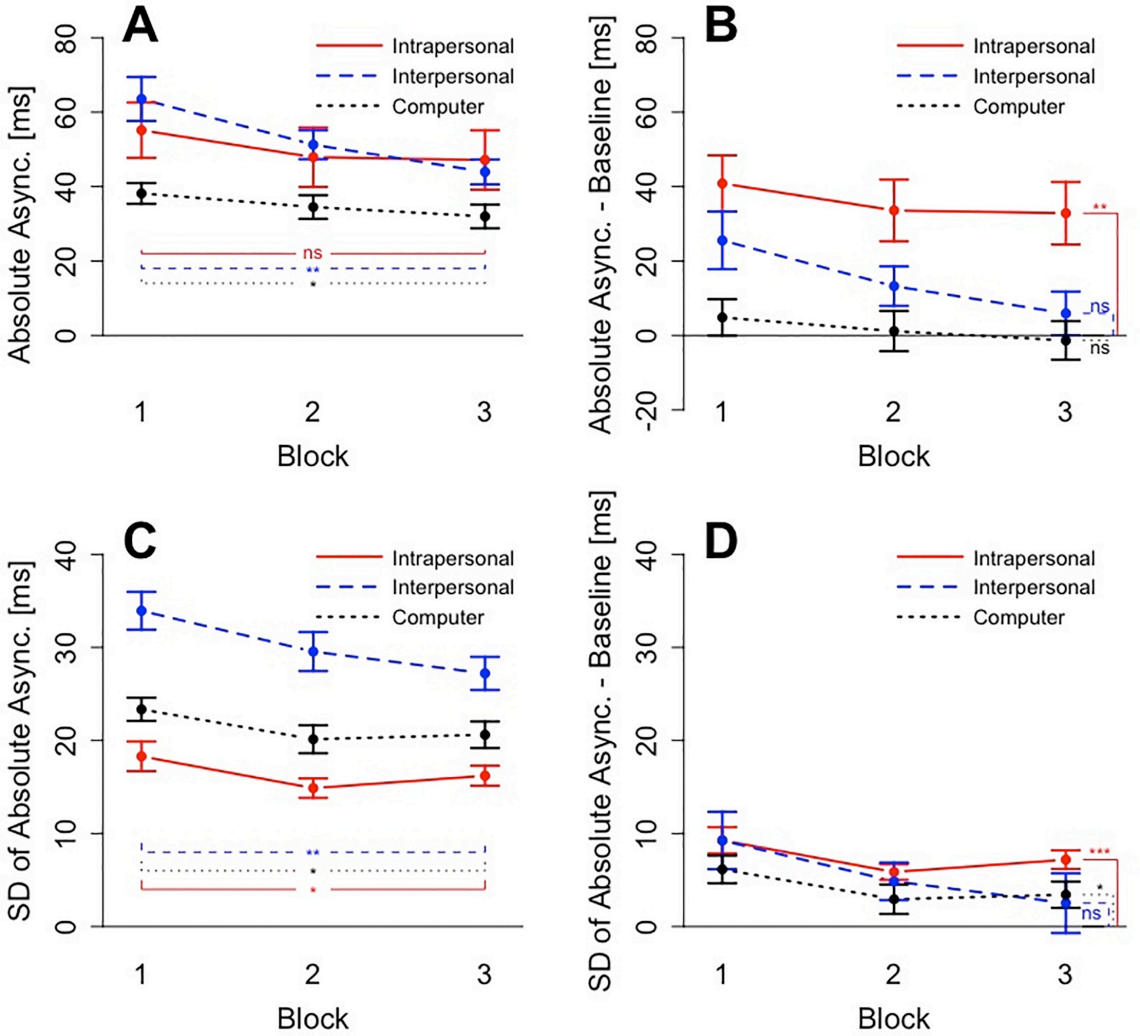
Results for delay trials of Experiment 1. All panels show Block on the x axis. Panel A) shows the results for absolute asynchrony. Panel B) also shows absolute asynchronies but corrected for the baseline from non-delay trials in each condition. Panel C) shows performance in terms of variability (SD) of absolute asynchrony. Panel D) shows baseline-corrected variability. Error bars in all four panels show standard errors.

To asses participants’ improvements across delay trials, we computed a 3 x 3 ANOVA with the factors Condition (Individual, Joint and Computer) and Block (One, Two and Three). The corresponding plot for absolute asynchronies can be seen in Fig 4 –Panel A. Degrees of freedom were corrected using Greenhouse-Geiser estimates of sphericity. The ANOVA revealed both a main effect of Condition, *F*(1.277, 14.050) = 5.531, *p* = .027, *η*^*2*^ = .159, and Block, *F*(1.435, 15.780) = 18.124, *p* < .001, *η*^*2*^ = .062, but no significant interaction *F*(2.044, 22.483) = 2.516, *p* = .102, *η*^*2*^ = .018. Post-hoc comparisons with Holm-Bonferroni corrected p-values revealed that the main effect of condition was driven by the lower absolute asynchrony in the Computer condition. There was no significant difference between the Intra- and the Interpersonal condition, *t*(11) = .500, *p* = .627, *d* = .144, nor between the Intrapersonal and the Computer condition, *t*(11) = 2.013, *p* = .208, *d* = .581. Compared to the Computer condition the asynchrony was higher in the Interpersonal condition, *t*(11) = 5.043, *p* = .002, *d* = 1.456. Post-hoc comparisons further revealed that the performance in the Intrapersonal condition in the first block (M = 55 ms, SD = 26 ms) and in the last block (M = 47 ms, SD = 28 ms) did not differ significantly, *t*(11) = 1.557, *p* = .295, *d* = .449, whereas the performance in the Interpersonal condition (M_First_ = 64 ms, SD_First_ = 20 ms, M_Last_ = 44 ms, SD_Last_ = 12 ms) improved significantly, *t*(11) = 4.747, *p* = .003, *d* = 1.370. There was also a significant improvement in the Computer condition (M_First_ = 38 ms, SD_First_ = 10 ms, M_Last_ = 32 ms, SD_Last_ = 11 ms), *t*(11) = 3.148, *p* = .037, *d* = .909. To, summarize, overall participants exhibited the lowest asynchronies in the Computer condition and showed significant improvement in the Computer and the Interpersonal condition.

A similar ANOVA was computed for baseline-corrected absolute asynchronies (see Fig 4 –Panel B). Degrees of freedom were corrected using Greenhouse-Geiser estimates of sphericity. The ANOVA revealed both a main effect of Condition, *F*(1.430, 15.725) = 8.386, *p* = .006, *η*^*2*^ = .289, and Block, *F*(1.434, 15.780) = 18.124, *p* < .001, *η*^*2*^ = .043, but no significant interaction *F*(2.044, 22.483) = 2.516, *p* = .102, *η*^*2*^ = .013. Post-hoc comparisons with Holm-Bonferroni corrected p-values revealed that in the first block, the baseline-corrected performance in the Interpersonal condition (M = 26 ms, SD = 27 ms) was not significantly different from the performance in the Intrapersonal condition (M = 40 ms, SD = 26 ms), *t*(11) = 2.074, *p* = .125, *d* = .599, and not different from the performance in the Computer condition (M = 5 ms, SD = 17 ms), *t*(11) = 2.402, *p* = .105, *d* = .693. Due to the improvement of the performance in the Interpersonal condition, this pattern was different for the last block, where the baseline-corrected performance in the Interpersonal condition (M = 6 ms, SD = 20 ms) was significantly lower than the performance in the Intrapersonal condition (M = 33 ms, SD = 29 ms), *t*(11) = 3.626, *p* = .016, *d* = 1.047, but not statistically different from the performance in the Computer condition (M = -1 ms, SD = 18 ms), *t*(11) = 1.022, *p* = .329, *d* = .295. When tested against 0 (here: equal to baseline), a one-sample t-test revealed that in the Computer condition participants performance was not significantly different from baseline performance already in the first block, *t*(11) = .983, *p* = .347, *d* = .284. In the Interpersonal condition, participants’ performance was significantly different from baseline in the first block, *t*(11) = 3.306, *p* = .007, *d* = .954, but not in the last block, *t*(11) = 1.008, *p* = .335, *d* = .291. To summarize, due to significant improvement participants reached their baseline performance in the Interpersonal and the Computer condition, but not in the Intrapersonal condition.

### Variability in baseline trials

As we turn to the variability of absolute asynchrony, we see a slightly different picture. In non-delay baseline trials we observed the lowest variability in the Intrapersonal condition (M = 3 ms, SD = 2 ms), with Interpersonal (M = 12 ms, SD = 10 ms) showing the highest variability and Computer (M = 6 ms, SD = 4 ms) falling in between (see Fig 3 –Panel B). We computed a one-way ANOVA for the factor Condition (Intrapersonal, Interpersonal and Computer). The Greenhouse-Geiser corrected ANOVA revealed a significant effect for Condition, *F*(1.168, 12.845) = 38.107, *p* < .001, *η*^*2*^ = .690. Post-hoc paired-samples t-tests with Holm-Bonferroni corrected p-values showed that variability in the Intrapersonal condition was significantly lower than in the Computer condition, *t*(11) = 11.416, *p* < .001, *d* = 3.296 and variability in the Computer condition was significantly lower than in the Interpersonal condition, *t*(11) = 3.584, *p* = .004, *d* = 1.035. To summarize, participants exhibited the lowest variability in the Intrapersonal condition.

### Variability in delay trials

To asses participants’ reduction of variability across delay trials, we computed a 3 x 3 ANOVA with the factors Condition (Intrapersonal, Interpersonal and Computer) and Block (1, 2, and 3). The corresponding plot for variability can be seen in Fig 4 –Panel C. Degrees of freedom were corrected using Greenhouse-Geiser estimates of sphericity. The ANOVA revealed both a main effect of Condition, *F*(1.478, 16.259) = 35.871, *p* < .001, *η*^*2*^ = .544, and Block, *F*(1.483, 16.313) = 11.853, *p* = .001, *η*^*2*^ = .104, but no significant interaction, *F*(1.908, 20.99) = 1.810, *p* = .189, *η*^*2*^ = .028. Post-hoc comparisons with Holm-Bonferroni corrected p-values showed that variability in the Intrapersonal condition was significantly lower than in the Computer condition, *t*(11) = 3.557, *p* = .013, *d* = 1.027, which was in turn significantly lower than the variability in the Interpersonal condition, *t*(11) = 6.401, *p* < .001, *d* = 1.848. Post-hoc comparisons furthermore revealed that the performance in the Intrapersonal condition in the first block (M = 18 ms, SD = 6 ms) and in the last block (M = 16 ms, SD = 4 ms) differed significantly, *t*(11) = 2.333, *p* = .04, *d* = .674, as did the performance in the Interpersonal condition (M_First_ = 34 ms, SD_First_ = 7 ms, M_Last_ = 27 ms, SD_Last_ = 6 ms), *t*(11) = 4.350, *p* = .005, *d* = 1.256. There was also a significant reduction of variability in the Computer condition (M_First_ = 23 ms, SD_First_ = 4 ms, M_Last_ = 21 ms, SD_Last_ = 5 ms), *t*(11) = 3.130, *p* = .019, *d* = .904. To summarize, participants exhibited the lowest variability in the Intrapersonal condition and showed significant improvement in all conditions.

A similar ANOVA was computed for baseline-corrected variability (see Fig 4 –Panel D). The Greenhouse-Geiser corrected ANOVA only revealed a main effect of Block, *F*(1.483, 16.313) = 11.853, *p* = .001, *η*^*2*^ = .070. Neither the main effect of Condition, *F*(1.258, 13.838) = 1.130, *p* = .323, *η*^*2*^ = .041, nor the interaction, *F*(1.908, 20.99) = 1.810, *p* = .189, *η*^*2*^ = .018, were significant. Comparing the baseline-corrected variability measures to zero in one-sample t-tests, showed that in the Intrapersonal condition even in the last block participants did not reach the baseline performance, *t*(11) = 7.146, *p* < .001, *d* = 2.063. There was also a significant difference between the performance in delay trials and the baseline in the Computer condition, *t*(11) = 2.423, *p* = 0.034, *d* = .699. However, the performance in the last block of the Interpersonal condition was not significantly different from baseline, *t*(11) = .783, *p* = 0.450, *d* = .226, indicating that baseline performance was reached in the Interpersonal condition. Hence, in terms of variability of asynchrony participants reached levels comparable to baseline performance only in the Interpersonal condition.

### Absolute asynchrony and its variability combined

Table 1 offers a detailed overview of individual differences among participants. Three participants managed to reach their baseline performance in at least one bin in each condition. Five more participants reached baseline performance in at least one bin in the Interpersonal and the Computer condition. Three participants managed to reach baseline only in the Computer condition and one participant did not reach their baseline in any of the three conditions.

**Table 1:** Overview of participants’ performance.

Experiment 1				Experiment 2			
ID	Intra-personal	Inter-personal	Computer	ID	Intra-personal	Inter-personal	Computer
1	0	4	6	1	0	4	0
2	0	0	4	2	0	5	6
3	0	3	4	3	0	6	6
4	0	1	6	4	1	1	0
5	0	0	1	5	0	6	6
6	0	0	0	6	0	0	5
7	1	6	4	7	0	2	6
8	1	6	2	8	0	6	5
9	3	6	6	9	0	3	4
10	0	0	5	10	0	1	0
11	0	1	1	11	0	0	1
12	0	3	4	12	0	1	6
	5	30	43		1	35	45

Number of bins (each bin was 10 trials / half a block) in which individual participants’ performance was comparable to their non-delay baseline performance, both, in terms of absolute asynchronies and its variability. There were 6 bins in each condition. The last row shows the column sums. Note that whereas in the group statistics participants never reached the baseline performance in the Intrapersonal condition, this table shows that there were six participants who managed to do so in all conditions, including the Intrapersonal condition (green fill color/dashed frame lines).

## Experiment 2 –Methods

### Participants

For the second experiment, we invited another 12 musicians to participate in the experiment (5 women, 7 men, mean age = 27 years, SD = 6 years). All 12 participants managed to produce 80% of the trials successfully. All participants in Experiment 2 had completed at least 5 years of private lessons on a musical instrument (M = 10 years, SD = 4 years). As in Experiment 1, information about statistical power was computed with G*Power [36]. As the sample size is the same as in Experiment 1, power for all 3-level within-subjects factors in 3x3 ANOVAs is the same for Experiment 2 as for Experiment 1, namely 0.82, 0.41 and 0.10 for large, medium and small effects correspondingly.

For the between comparison of Experiment 1 and 2, we used data from all 24 participants and entered them into a 2x3 mixed ANOVA. Based on the total sample size of 24, power for the 3-level within-subjects factor in the mixed design corresponds to 0.99, 0.74 and 0.16, for large, medium and small effect sizes. Power for the 2-level between-subjects factor in the mixed design for the comparison across experiments corresponds to 0.63, 0.30 and 0.09 for large, medium and small effect sizes.

### Procedure and design

Procedure and design were almost identical to Experiment 1. As in Experiment 1, the 12 musicians were grouped into 6 pairs for the Interpersonal condition. In Experiment 2, the actions participants had to perform were exactly the same as in Experiment 1. Only the outcomes that were produced by their actions were different. In Experiment 2 all participants’ taps, as well as the computer metronome, produced piano sounds of different pitches that taken together generated a short polyphonic melody of ten beats (see Fig 1). Hence, action outcomes exhibited an additional structure of harmonic pitch. In short, in Experiment 2 the actions were identical to those in Experiment 1, but the produced outcomes differed from those in Experiment 1.

## Experiment 2 –Results

### Accuracy

The mean accuracy for the participants in Experiment 2 was 94.91% (SD = 3.50%). A Greenhouse-Geiser corrected one-way ANOVA with the Factor Condition revealed a significant main effect, *F*(1.208, 13.289) = 4.753, *p* = .042, *η*^*2*^ = .22. However, post-hoc comparisons with Holm-Bonferroni corrected p-values revealed no significant differences. The accuracy was highest in the Computer condition (M = 98.31%, SD = 2.51%), but not significantly different from both the accuracy in the Intrapersonal condition (M = 90.97%, SD = 9.52%), *t*(11) = 2.623, *p* = .058, *d* = .757, and the accuracy in the Interpersonal condition (M = 95.44%, SD = 3.00%), *t*(11) = 2.737, *p* = .058, *d* = .79. Furthermore, there was no significant difference between Intrapersonal and Interpersonal condition, *t*(11) = 1.546, *p* = .150, *d* = .446. Hence, as in Experiment 1, there was no significant difference among the conditions in terms of accuracy.

### Asynchrony in baseline trials

In non-delay baseline trials in Experiment 2, we observed the lowest absolute mean asynchrony for the Intrapersonal condition (M = 14 ms, SD = 3 ms), with comparable levels of asynchrony for Interpersonal (M = 37 ms, SD = 8 ms) and Computer condition (M = 40 ms, SD = 15 ms) (see Fig 3 –Panel C). We computed a one-way ANOVA with the factor condition (Intrapersonal, Interpersonal and Computer). The Greenhouse-Geiser corrected ANOVA revealed a significant effect for Condition, *F*(1.772, 19.496) = 29.94, *p* < .001, *η*^*2*^ = .603. Post-hoc paired-samples t-tests with Holm-Bonferroni corrected p-values showed that this was mainly due to the lower asynchrony in the Intrapersonal condition, as compared to the Interpersonal *t*(11) = 7.563, *p* < .001, *d* = 2.183, and the Computer condition, *t*(11) = 6.205, *p* < .001, *d* = 1.791. There was no significant difference between the Interpersonal and the Computer condition, *t*(11) = .828, *p* = .425, *d* = .239. Hence, as in Experiment 1, when there was no delay participants exhibited lowest asynchronies in the Intrapersonal condition.

### Asynchrony in delay trials

**Fig 5:**
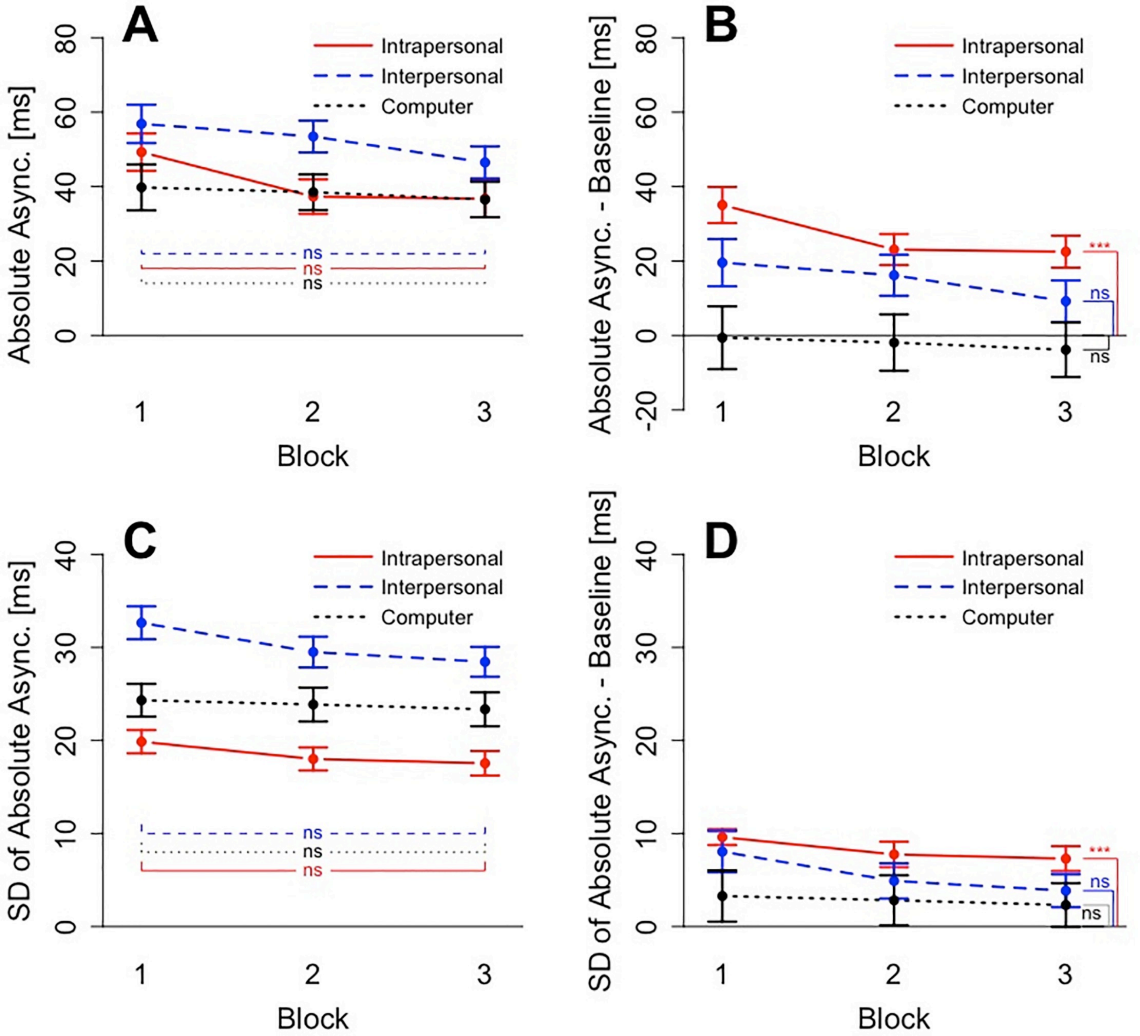
Results for delay trials of Experiment 2. Panel A) shows the results for absolute asynchrony. Panel B) also shows absolute asynchronies but corrected for the baseline from non-delay trials. Panel C) shows performance in terms of variability (SD) of absolute asynchrony. Panel D) shows baseline-corrected variability. Error bars in all four panels show standard errors.

Absolute asynchronies are displayed in Fig 5 –Panel A. A Greenhouse-Geiser corrected 3 x 3 ANOVA with the factors Condition (Intrapersonal, Interpersonal and Computer) and Block (1, 2, and 3) revealed only a main effect of Block, *F*(1.242, 13.666) = 6.71, *p* = .017, *η*^*2*^ = .046, but no effect of Condition, *F*(1.712, 18.827) = 2.894, *p* = .087, *η*^*2*^ = .12, and no significant interaction effect, *F*(2.784, 30.626) = 2.165, *p* = .116, *η*^*2*^ = .018. Post-hoc comparisons with Holm-Bonferroni corrected p-values revealed that in contrast to the results in Experiment 1, in Experiment 2 there was no significant improvement. Asynchronies in the Intrapersonal condition was higher in the first block (M = 49 ms, SD = 17 ms) than in the last block (M = 37 ms, SD = 17 ms), *t*(11) = 2.325, *p* = .080, *d* = .671. The performance in the Interpersonal condition also improved, albeit not significantly (M_First_ = 57 ms, SD_First_ = 18 ms, M_Last_ = 46 ms, SD_Last_ = 15 ms), *t*(11) = 2.679, *p* = .064, *d* = .773 and there was no significant improvement in the Computer condition (M_First_ = 40 ms, SD_First_ = 21 ms, M_Last_ = 37 ms, SD_Last_ = 16 ms), *t*(11) = 1.448, *p* = .176, *d* = .418. This suggests that there was an overall effect of Block, which did not manifest in separate improvement for any one of the conditions.

A similar ANOVA was computed for baseline-corrected absolute asynchronies (see Fig 5 –Panel B). The Greenhouse-Geiser corrected ANOVA revealed a significant main effect of Condition, *F*(1.652, 18.169) = 6.998, *p* = .008, *η*^*2*^ = .252, and a significant main effect of Block, *F*(1.242, 13.666) = 6.71, *p* = .017, *η*^*2*^ = .030. The interaction, however, was not significant, *F*(2.784, 30.626) = 2.165, *p* = .116, *η*^*2*^ = .012. Post-hoc t-tests with Holm-Bonferroni corrected p-values showed that the baseline-corrected performance in the Interpersonal condition was neither significantly different from the Intrapersonal condition, *t*(11) = 2.043, *p* = .122, *d* = .59, nor from the Computer condition, *t*(11) = 2.083, *p* = .122, *d* = .601. Asynchronies in the Intrapersonal condition, however, were significantly higher than in the Computer condition, *t*(11) = 3.224, *p* = .024, *d* = .931. Comparing the baseline-corrected values to zero in one-sample t-tests, showed that, as in Experiment 1, participants reached baseline performance in the last block of the Interpersonal condition, *t*(11) = 1.643, *p* = 0.129, *d* = .474, and already in the first block in the Computer condition, *t*(11) = .071, *p* = 0.945, *d* = .020. However, in the last block of the Intrapersonal condition, performance was still significantly higher than baseline, *t*(11) = 5.232, *p* < .001, *d* = 1.51. This means that in the Computer and the Interpersonal condition, but not in the Intrapersonal condition, participants reached levels of asynchrony which are comparable to their baseline performance.

### Variability in baseline trials

In non-delay baseline trials we observed the lowest variability in the Intrapersonal condition (M = 10 ms, SD = 3 ms), with higher variability in the Interpersonal condition (M = 25 ms, SD = 4 ms) and in the Computer condition (M = 21 ms, SD = 6 ms) (see Fig 3 –Panel D). We computed a one-way ANOVA with the factor Condition (Intrapersonal, Interpersonal and Computer). The Greenhouse-Geiser corrected ANOVA revealed a significant effect for Condition, *F*(1.937, 21.304) = 37.596, *p* < .001, *η*^*2*^ = .658. Post-hoc paired-samples t-tests with Holm-Bonferroni corrected p-values showed that this was due to the significant difference between the Intrapersonal condition and both the Interpersonal condition, *t*(11) = 8.145, *p* < .001, *d* = 2.351, and the Computer condition, *t*(11) = 5.892, *p* < .001, *d* = 1.701, and a significant difference between the Interpersonal condition and the Computer condition, *t*(11) = 2.273, *p* = .044, *d* = .656. Hence, as in Experiment 1, participants exhibited their lowest asynchronies in non-delay trials in the Intrapersonal condition.

### Variability in delay trials

We computed a 3 x 3 ANOVA with the factors Condition (Intrapersonal, Interpersonal and Computer) and Block (One, Two, and Three). The corresponding plot can be seen in Fig 5 –Panel C. The Greenhouse-Geiser corrected ANOVA revealed a significant main effect of condition, *F*(1.818, 20.000) = 21.282, *p* < .001, *η*^*2*^ = .450, and a significant main effect of block, *F*(1.950, 21.451) = 5.737, *p* = .011, *η*^*2*^ = .038. The interaction was not significant, *F*(2.193, 24.120) = .757, *p* = .491, *η*^*2*^ = .012. Post-hoc t-tests with Holm-Bonferroni corrected p-values revealed that the variability in the Intrapersonal condition was not significantly lower than the variability in the Computer condition, *t*(11) = 2.601, *p* = .099, *d* = .751, which in turn was significantly lower than the variability in the Interpersonal condition, *t*(11) = 7.18, *p* < .001, *d* = 2.073. Post-hoc comparisons revealed furthermore that the performance in the Intrapersonal condition in the first block (M = 20 ms, SD = 4 ms) and in the last block (M = 18 ms, SD = 5 ms) did not differ significantly, *t*(11) = 2.020, *p* = .137, *d* = .583., nor was there a significant reduction of variability in the Interpersonal condition (M_First_ = 33 ms, SD_First_ = 6 ms, M_Last_ = 28 ms, SD_Last_ = 6 ms), *t*(11) = 2.480, *p* = .099, *d* = .716, nor in the Computer condition (M_First_ = 24 ms, SD_First_ = 6 ms, M_Last_ = 23 ms, SD_Last_ = 6 ms), *t*(11) = .619, *p* = .548, *d* = .179. This means, that the significant main effect of Block was not due to any individual condition in particular.

A similar ANOVA was computed for baseline-corrected variability (see Fig 5 –Panel D). The Greenhouse-Geiser corrected ANOVA for the second experiment revealed a significant main effect of block, *F*(1.950, 21.451) = 5.737, *p* = .011, *η*^*2*^ = .024, but not of condition, *F*(1.746, 19.202) = 2.810, *p* = .091, *η*^*2*^ = .099. The interaction was also not significant, *F*(2.193, 24.120) = .757, *p* = .491, *η*^*2*^ = .008. Post-hoc comparisons with Holm-Bonferroni corrected p-values revealed no significant difference between the Block One and Block Three in the Interpersonal condition, t(11) = 2.48, p = .092, d = .716, nor in the Intrapersonal condition, *t*(11) = 2.020, *p* = .137, *d* = .583, nor in the Computer condition, *t*(11) = .619, *p* = .548, *d* = .179. Comparing the baseline-corrected variability measures to zero in one-sample t-tests, showed that even in the last block participants had a performance significantly worse than baseline, in the Intrapersonal condition, *t*(11) = 5.461, *p* < .001, *d* = 1.576. However, the performance in the last block of the Interpersonal condition, *t*(11) = 2.179, *p* = 0.052, *d* = .629 and of the Computer condition, *t*(11) = 1.190, *p* = 0.259, *d* = .344, was not significantly different from baseline performance. As in Experiment 1, participants reached their baseline performance in terms of variability of asynchronies in the Interpersonal condition.

### Absolute asynchrony and its variability combined

In Experiment 2, there was no participant who reached their baseline performance in all three conditions (see Table 1). Nine participants reached their baseline in two conditions and three participants only in one condition.

## Cross-experiment comparison

### Adaptation performance across experiments

In Experiment 1 participants reached their baseline performance on average in 2.5 of 6 bins in the Interpersonal condition and on average 3.6 of 6 bins in the Computer condition. In Experiment 2, these numbers are 2.9 of 6 bins and 3.8 of 6 bins respectively. A Greenhouse-Geiser corrected 3 x 2 ANOVA on this data with the within-subjects factor Condition (Intrapersonal, Interpersonal, Computer) and the between-subjects factor Experiment (1 and 2) revealed a main effect for Condition, *F*(1.918, 42.186) = 24.507, *p* < .001, *η*^*2*^ = .357, but no effects for Experiment, *F*(1, 22) = .020, *p* = .888, *η*^*2*^ = .000, and no significant interaction, *F*(1.918, 42.186) = .288, *p* = 0.742, *η*^*2*^ = .006.

To establish whether an effect of Experiment would only manifest itself in either asynchronies or variability thereof, we also calculated 3x3x2 ANOVAs with the factors Condition, Block and Experiment for all four dependent variables separately. While the main effects for Condition and Block were significant (all p-values < .03) the main effects for Experiment were not (all p-values > .27).

### Questionnaire data

All participants provided us with some biographic data in a post-experiment questionnaire. Besides standard items such as age and handedness, we asked which instruments they played, for how long they had training on them, whether they had experience playing in ensembles or teaching their instrument. To see whether any of these items are good predictors for their performance in the adaptation task, we entered them in a linear regression model as predictors for how many delayed bins where performed at baseline levels. We started with a model that contained the following four predictors: *age*, *main training* (the number of years they had received training on their main instrument), *total training* (the number of years they had received training on any instrument aggregated over all instruments they had provided us with data, e.g. for a participant that had 19 years of training on the piano and 9 years on the flute, *main training* was coded as 19, whereas *total training* was coded as 28) and *practice per week* (in hours). We systematically removed the factor with the highest p-value until only significant predictors were left [38]. This left us with the two significant predictors *age* and *main training*. Whereas an increase in *main training* predicted better performance, an increase in *age* predicted worse performance (see Table 2 for further details). As assumptions of normality were not met, we validated this model with a boot strapping procedure [38] that confirmed the significant effects of *age* and *main training*.

**Table 2:** Linear regression model with questionnaire data.

	Model Stats	Estimate	Standard Error	p-value	95% Confidence Intervals		95% Confidence Intervals (Boot strapping)	
*intercept*	R^2^ = 0.210 (adjusted) p = .032	12.438	4.170	0.007	2.388	16.925	3.890	19.920
*age*		-0.397	0.166	0.026	-0.742	-0.052	-0.660	-0.140
*main training*		0.428	0.190	0.035	0.032	0.824	0.010	0.827

Results for the linear model *successful bins ~ age + main training + error*. For the bootstrapping procedure we used 2000 iterations. *Age* was coded as the age of participants at the time of the experiment. *Main training* was coded as the amount of years each participant had on their main instrument.

## Discussion

In this study we investigated the effects of coordination setting and structure of action outcomes on musicians’ ability to adapt to unstable coordination patterns. First, we tested conditions implying various degrees of coupling strength between two movements. For non-delay baseline performance, we found a clear pattern that repeated across both experiments. Absolute asynchrony was significantly lower and more stable in the Intrapersonal condition than in the Interpersonal condition, which is in line with findings from studies that compare intrapersonal and interpersonal coordination [39]. This pattern is probably due to the lack of shared internal processes [39] in interpersonal coordination and the beneficial effects of stronger coupling on in-phase coordination in intrapersonal coordination [3–5]. In the unidirectional coupling condition where participants performed with a computer, they exhibited absolute asynchronies to the same degree as in the interpersonal condition. Konvalinka and colleagues [40] argued on the basis of similar results that a less predictable, but responsive partner facilitates synchronization just as much as a perfectly predictable, but un-responsive computer. However, in the current study we found that variability of asynchrony was consistently better in the Computer condition than in the Interpersonal condition. This suggests that the asynchrony in the Computer condition arose to some extent from a more stable type of coordination and is most likely based on the well-established negative mean asynchrony [41].

Whereas participants’ baseline performance was best in the Intrapersonal condition, we predicted that the weaker coupling in the Interpersonal condition and the Computer condition should be advantageous for the performance of unstable coordination patterns. In terms of absolute asynchrony, for which we saw a clear advantage of stronger coupling during in-phase coordination, we find that this advantage disappeared during the performance of an unstable coordination pattern. In Experiment 1, asynchrony was lowest in the Computer condition and equal in the Intrapersonal and Interpersonal condition. In Experiment 2, we found no differences between the three coordination conditions. In Experiment 1, the improvement of asynchrony performance from the first to the last block was not significant in the Intrapersonal condition, whereas it was significant for the Interpersonal condition. For asynchrony in the Interpersonal condition, on the other hand, we see significant improvement across blocks in Experiment 1. In Experiment 2 there was a general effect of Block. While none of the individual conditions showed significant improvement after p-value corrections, participants reached their baseline performance only in the Interpersonal and the Computer condition. The effects of an unstable coordination pattern in the different coordination conditions was clearly visible in comparison to the baseline. In the Computer condition, where the coupling was just unidirectional, we saw that participants managed to reach the same performance as in their baseline already in Block 1 in both experiments. In the Interpersonal condition, participants initially performed significantly worse than their baseline. However, thanks to continuous adaptation across the three blocks, participants eventually reached their baseline performance in Experiment 1 and 2. In the Intrapersonal condition participants never reached their baseline performance. Hence, in terms of absolute asynchrony, we conclude that stronger intrapersonal coupling impedes coordination of, and adaptation to, unstable patterns.

While in absolute terms the variability of asynchrony in delay trials was lowest in the Intrapersonal condition, baseline corrected variability showed that participants were on average not able to reach their baseline performance. In contrast, in the Interpersonal condition baseline performance was reached in Experiment 1 and Experiment 2. Taking together asynchrony and its variability, we found that during the Intrapersonal Condition, the condition with the strongest coupling, the group average never reached the baseline performance, even though we saw significant reduction in variability for Experiment 1. It is interesting to note that the Intrapersonal condition was also the condition with the consistently lowest variability. Individual motor learning studies have shown that variability is advantageous for motor learning [42,43]. This leads to the question of whether adaptation in the Interpersonal condition was facilitated by the higher variability in that condition. Whether high variability in joint tasks leads to better motor learning is an important question for research on coordination in joint action and is currently under investigation by Sabu and colleagues [44].

Taken together, the results discussed so far show that reducing the coupling strength between interacting limbs by the means of splitting a task across two people might facilitate the production of otherwise unstable coordination patterns. This principle is reminiscent of Ugandan xylophone players who manage to produce unstable coordination patterns at incredibly high tempi by distributing them across two musicians while keeping in-phase actions within musicians.

In cultural evolution, where intending patterns, retaining them and reproducing them is limited by cognitive constraints [45,46] and possibly also by physical constraints [47], copying errors should lead to a convergence towards in-phase patterns over time [48]. To use the terminology of the cultural attraction theory, in-phase dominance [see e.g. 49] could be a factor of attraction and if so, it would be a global factor, not limited to a certain population or time [48]. However, instead of a convergence, we see a variety of musical contexts that deviate from in-phase coordination patterns. Recent transmission chain experiments showed that one-part rhythmic patterns converged to exhibit structural organization that was based on small-integer ratios [see e.g. 50]. Whether two-part rhythmic patterns would also converge on small-integer ratios seems likely but remains to be tested empirically. This is also the case for possible differences between transmission chains in which two-part patterns are produced bimanually and chains in which patterns are produced jointly. Hence, the question of whether intricate rhythms in the evolution of music initially emerged out of joint music-making rather than solo music-making remains to be answered by further research.

As a second factor we investigated the effects of structure realized along the harmonic pitch dimension on the adaptation to unstable coordination patterns. We predicted better adaptation in Experiment 2, where the auditory outcome was supplemented by a melodic structure. In terms of how often participants accomplished performance in delay trials that was comparable to their baseline, the data exhibited the effect of coordination setting, but no differences between the results of the two experiments. Hence, the melodic enhancement of the auditory outcome did not lead to significantly better adaptation. Because we needed to recruit experienced musical experts for our study our small sample size was relatively small. Therefore, we cannot exclude the possibility of having missed effects with small to medium effect sizes. Thus, further studies might be needed to investigate the effects of added structure in the production of unstable coordination patterns and its relation to sonification effects in bimanual motor skill learning [32].

Given that musicians frequently encounter unstable coordination patterns, we had expected the invited musicians to more readily adapt to the pattern than the data suggested. As mentioned above, our subjects had on average around 11 years of musical training. Maybe this is not enough training to build the necessary skills to deploy similar strategies like the third player in Amadinda music, who has to be able to use structured action effects on which to map the necessary motor commands. However, the data show that at least some participants were in fact able to adapt to the strange phase relation in all conditions, especially in the Computer and the Interpersonal condition, but also in the Intrapersonal condition, where coupling was strongest.

In the Intrapersonal condition of Experiment 1, three participants reached a delay performance that was not significantly different from their non-delay baseline, both in terms of absolute asynchrony as well as its variability. In the Intrapersonal condition in Experiment 2, only one participant managed to reach their baseline performance. Across both experiments, there were six participants who reached the baseline performance within the 3 blocks in all three condition, including the Intrapersonal condition for which the group average never reached the baseline. One participant did not manage to reach their baseline performance in any of the coordination conditions.

To identify possible predictors of success in terms of reaching one’s baseline performance, we used a linear model procedure on collected questionnaire data, from which two significant predictors emerged: age and years of training on the main instrument. *Years of training* was positively correlated with success, which suggests that experience as a musician improves the ability to adapt to unstable phase relations. Age, however, was negatively correlated with success. The fact that the model accounted for less than 20% of the variability suggests that there are other factors that contribute to the ability to adapt to unstable coordination patterns. The effect of years of training could, for example, be mediated by instrument type and type of training. Some instruments might require more temporal flexibility than other instruments, such as drums and piano, on which playing different rhythmic patterns at the same time is frequent. Experience in mixed ensembles with instruments that exhibit different rise times might also train the ability to adapt to unstable coordination patterns.

It might also be noteworthy that participant 9 in Experiment 1, who adapted well to all three conditions, had eight years of experience as an organ player. Gould and Helder [51] reported that the only person who was able to speak coherently in a speech delay experiment that they had conducted was a professional organist. As mentioned in the introduction, pipe organs can exhibit pitch-dependent delays of up to 150 ms. This could mean that some organists are especially trained in flexibly mapping different delays to different finger actions in order to align tone onsets. However, the current study does not allow us to address the role of musical training in the production of unstable coordination patterns.

For further research on unstable coordination patterns in joint actions, it would be interesting to identify the skills or strategies that allowed some participants to quickly adapt to the phase shift. Following up on this it would also be interesting to investigate whether these skills or strategies are transferable to other unstable coordination patterns and also whether non-musicians would be able to learn these skills and/or strategies isolated from musical training. Another possible avenue for further research in this direction would lead towards an understanding of the influence of joint music-making as a tool to avoid certain attractor states, such as in-phase coordination in the transmission of culture.
